# Meta-analysis of the prevalence of anxiety and depression among frontline healthcare workers during the COVID-19 pandemic

**DOI:** 10.3389/fpubh.2022.984630

**Published:** 2022-09-13

**Authors:** Yu Chen, Jing Wang, Yujie Geng, Zhengmei Fang, Lijun Zhu, Yan Chen, Yingshui Yao

**Affiliations:** ^1^School of Public Health, Wannan Medical College, Wuhu, China; ^2^Department of Clinical Medicine, Anhui College of Traditional Chinese Medicine, Wuhu, China

**Keywords:** COVID-19, frontline healthcare workers, anxiety, depression, prevalence, meta-analysis

## Abstract

**Objective:**

To systematically review the prevalence of anxiety and depression among frontline healthcare workers during the coronavirus disease 2019 (COVID-19) pandemic.

**Methods:**

Computers were used to search CNKI, VIP, WanFang Data, PubMed, and other Chinese and English databases. The search period was limited to December 2019 to April 2022. Cross-sectional studies collected data on the prevalence of anxiety and depression among frontline healthcare workers since the onset of COVID-19. The STATA 15.1 software was used for the meta-analysis of the included literature.

**Results:**

A total of 30 studies were included, with a sample size of 18,382 people. The meta-analysis results showed that during the COVID-19 pandemic, the total prevalence of anxiety among frontline healthcare workers was 43.00%, with a 95% confidence interval (CI) of 0.36–0.50, and the total prevalence of depression was 45.00%, with a 95% CI of 0.37–0.52. The results of the subgroup analysis showed that prevalence of anxiety and depression in women, married individuals, those with children, and nurses was relatively high. Frontline healthcare workers with a bachelor's degree or lower had a higher prevalence of anxiety. The prevalence of depression was higher among frontline healthcare workers with intermediate or higher professional titles.

**Conclusion:**

During the COVID-19 pandemic, the prevalence of anxiety and depression among frontline healthcare workers was high. In the context of public health emergencies, the mental health status of frontline healthcare workers should be given full attention, screening should be actively carried out, and targeted measures should be taken to reduce the risk of COVID-19 infection among frontline healthcare workers.

**Systematic review registration:**

http://www.crd.york.ac.uk/PROSPERO/, identifier: CRD42022344706.

## Introduction

At the end of 2019, the corona virus disease 2019 (COVID-19) caused by the SARS-CoV-2 virus, appeared and rapidly developed into a major international public health emergency. In the early days of the outbreak, because of the high risk, contagiousness, and lack of awareness about COVID-19, many areas stopped production and work and adopted home isolation measures to reduce the spread of the virus and the risk of infection. After unremitting efforts, China is currently in the stage of normalized prevention and control of the new crown pneumonia (1–3).

Since the outbreak of COVID-19, healthcare workers have been the core force in fighting the pandemic and are also a high-risk group for viral infection. Among them, frontline healthcare workers must always face the virus directly, mostly in a high-pressure environment, to carry out rescue and other work. People have exhibited a trend of increased mental health issues during the COVID-19 pandemic (4). Due to this type of work, the mental health of frontline healthcare workers may be more vulnerable. At the beginning of the pandemic, owing to a lack of relevant experience, anti- pandemic materials, and effective treatment methods, frontline healthcare workers were under enormous psychological pressure (5, 6). Therefore, attention should be paid to the mental health of healthcare workers, especially frontline workers. By providing them with targeted assistance and enlightenment, their psychological burden can be reduced. This will not only maintain frontline healthcare workers' mental health but will also help patients receive better treatment, and is thus, conducive to the development of follow-up pandemic prevention and control work (7, 8). Therefore, it is necessary to explore the mental health status and negative emotions of frontline healthcare workers during COVID-19, including the prevalence of anxiety and depression. This could provide data to help control the global COVID-19 pandemic (9).

Among the various possible psychological problems of frontline healthcare workers, anxiety and depression are the most common, and the situation is similar to previous public health emergencies encountered by such workers (10–12). Among healthcare workers, an existing meta-analysis (13) showed that during the COVID-19 period, the prevalence of anxiety and depression was 24.94 and 24.83%, respectively, while another meta-analysis (6) evaluated the same prevalence in designated hospitals during the COVID-19 period, which was 44 and 31%, respectively. However, there has been no meta-analysis of such prevalence among frontline healthcare workers since the COVID-19 outbreak. Therefore, this study focused on the prevalence of anxiety and depression among frontline healthcare workers.

According to other studies, differences in gender and occupation may affect the prevalence of anxiety and depression (14). Based on these two possible factors, this study adds other potential factors to the analysis. The aim was to obtain additional results to provide a reference for subsequent targeted interventions.

This study systematically evaluated the risk of anxiety and depression among frontline healthcare workers during the COVID-19 pandemic. We further explored the possible potential influencing factors. The results can guide the the allocation of mental health services for healthcare workers and the implementation of targeted interventions. Thereby, reducing the prevalence of anxiety and depression amid the COVID-19 pandemic.

## Materials and methods

### Search strategy

A computer search of the CNKI, VIP, WanFang Data, and PubMed databases collected cross-sectional studies on the prevalence of anxiety and depression among frontline healthcare workers since the onset of COVID-19. The search period was limited to December 2019 to April 2022. A combination of subject headings and free words were used to search, and the search terms included COVID-19, anxiety, depression, healthcare workers, etc.

### Inclusion and exclusion criteria

The inclusion criteria were as follows: (1) research objects: frontline healthcare workers since the onset of COVID-19, (2) type of research: cross-sectional study, (3) research outcome: sample size and prevalence of anxiety and depression; and (4) language: Chinese or English. The exclusion criteria were as follows: (1) the research subject is part of other hospital populations, including non-frontline healthcare workers, administrative staff, interns, patients, and infected healthcare workers, (2) the same research or literature published repeatedly, (3) incorrect or incomplete data or inability to extract or convert required data, (4) lack of measurement tools featuring good reliability and validity used in assessing anxiety and depression; and (5) the research period did not coincide with the COVID-19 pandemic.

### Data extraction

Literature screening, data extraction, and cross-checking were performed independently by two trained researchers. Disagreements and doubts were resolved through three or more collective discussions or by consulting third-party experts. By reading the titles and abstracts, the literature that did not meet the requirements was initially excluded, and then the full texts of the remaining literature were read to determine whether they met the inclusion criteria. If the data were incomplete, we contacted the authors to obtain complete data. The extracted data mainly included the following: (1) basic information of the included studies, including the title, first author, time, and region of the study, (2) general information of the research subjects: sample size, age, sex, etc., (3) outcome indicators and outcome measurement data; and (4) key elements required for quality evaluation.

### Quality evaluation

The American Agency for Healthcare Research and Quality recommends quality evaluation standards for observational studies. The labels recommended for evaluating cross-sectional studies include 11 items, with responses “yes,” “no,” and “not reported” respectively. Only the answer “yes” scored 1, while “no” and “not reported” scored 0. Scores of 8–11 were regarded as high quality and 4–7 as moderate quality (15). Two researchers independently evaluated and cross-checked the quality of the included studies. In cases of disagreement, a third party was requested to assist in the discussion.

### Statistical methods

Meta-analysis was performed on the collected data using STATA 15.1 software; the prevalence of anxiety and depression and their 95% confidence interval (CI) were used as statistical effect sizes. The χ^2^ test (test level: α = 0.1) was used to analyze the heterogeneity among the results of the included studies, and the size of the heterogeneity was judged in combination with *I*^2^. Different heterogeneity models were used: (1) when *I*^2^ <50% and *P* > 0.10, the study was considered to be homogeneous, and the fixed effect model was used, (2) when *I*^2^ ≥ 50% and *P* ≤ 0.10, the study was considered to be heterogeneous, and a random effect model was used (16). Publication bias was detected using Egger's method. Publication bias refers to the fact that statistically significant research findings are more likely to be reported and published than non-significant and invalid findings (17). The stability and accuracy of the analysis results were evaluated using sensitivity analysis. It was used to assess the reliability of the meta-analysis results, focusing on study characteristics (such as the level of methodological quality). We excluded low-quality studies or adopted different efficacy evaluation standards and statistical methods to explore their influence on the combined effect size. The focus was on the comparison of the pooled effect sizes from repeated meta-analyses with the original effect sizes (18). *P* < 0.05 indicated that the difference was statistically significant.

## Results

### Selection of studies

**Figure 1:**
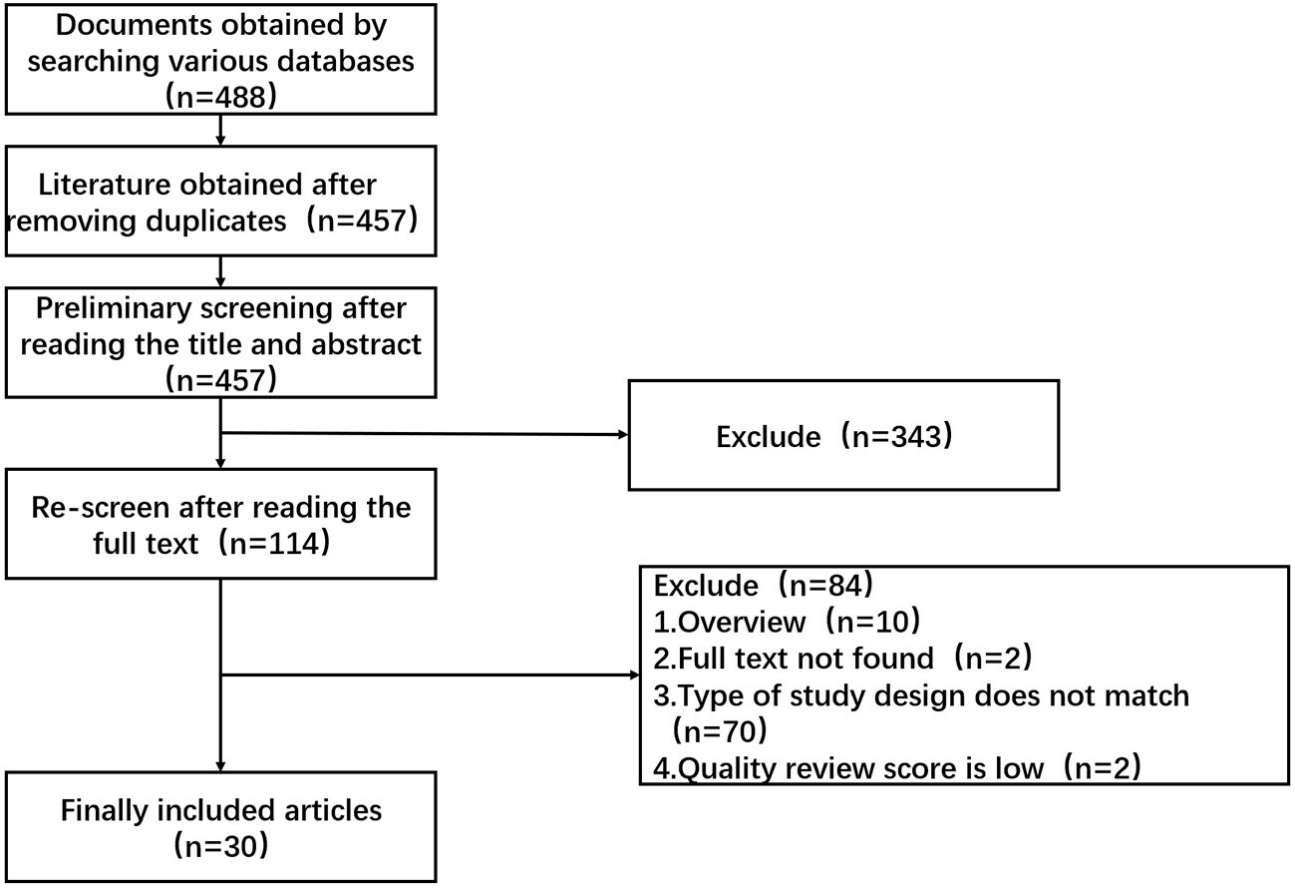
Literature screening process and results.

A total of 488 relevant studies were initially screened; after screening according to the inclusion and exclusion criteria, 30 studies were finally included (19–48). The literature screening process is illustrated in Figure 1.

### Basic characteristics

The basic characteristics of the included studies are presented in Table 1. The quality evaluation results showed that all included studies were of medium quality and above, including 3 high-quality articles and 27 medium-quality articles. Further details are provided in Table 2.

**Table 1:** Basic characteristics of included studies.

**Included studies**	**Country or region**	**Study sample size**	**Age of study subjects (years)**	**time of data collection (year/month/day)**	**Outcomes**	**Anxiety measurement tool**	**Depression measurement tool**	**Prevalence of anxiety [n (%)]**	**Prevalence of depression [n (%)]**	**AHRQ total score**
Hu et al. (19)	China	196	21–40	2020/1/24–2020/2/25	Anxiety	SAS		82(41.8)		7
Zhou et al. (20)	China	1,426	NR	2020/5/25 –2020/5/29	Anxiety, depression	SAS	SDS	194(13.6)	395(27.7)	4
Li et al. (21)	China	130	23–48	2020/2/6–2020/2/20	Anxiety, depression	DASS-21	DASS-21	101(77.69)	34(26.15)	6
Liu et al. (22)	China	69	22–44	2020/2/19–2020/2/20	Anxiety	DASS-21		44(63.77)		6
Xu et al. (23)	China	251	20–59	2020/2/4–2020/2/14	Anxiety, depression	SAS	SDS	62(24.70)	83(33.07)	5
Mei et al. (24)	China	105	NR	NR	Anxiety, depression	GAD-7	PHQ-9	19(46.67)	52(49.52)	4
Ma et al. (25)	China	128	23–56	NR	Anxiety, depression	GAD-7	PHQ-9	87(67.96)	119(92.96)	4
Guo et al. (26)	China	91	18–58	2020/2	Anxiety, depression	SAS	SDS	29(31.9)	59(64.9)	8
Chu et al. (27)	China	8316	NR	2020/7–2020/10	Anxiety, depression	GAD-7	PHQ-9	4,286(51.54)	4,619(55.54)	7
Zhao et al. (28)	China	380	22–59	2020/2/22–202/2/29	Anxiety, depression	GAD-7	PHQ-9	195(51.3)	256(67.4)	5
Tan et al. (29)	China	90	21–48	2020/1/31–2020/2/2	Anxiety, depression	SAS	SDS	33(36.67)	46(51.11)	5
Yuan et al. (30)	China	288	NR	NR	Anxiety, depression	SAS	SDS	155(53.82)	209(72.57)	7
Lei et al. (31)	China	148	18–60	2020/1/25–2020/2/29	Anxiety, depression	SAS	SDS	19(12.84)	56(37.84)	5
Huang et al. (32)	China	230	80–59	2020/2/7–2020/2/14	Anxiety	SAS		53(23.04)		5
Gong et al. (33)	China	174	36.38 ± 8.52	2020/1/26–2020/2/11	Anxiety, depression	HADS	HADS	51(29.31)	46(26.44)	4
Labrague and De Los Santos (34)	Philippines	325	30.94	2020/4/25–2020/5/25	Anxiety	The COVID-19 Anxiety Scale		123(37.8)		4
An et al. (35)	China	1,103	NR	2020/3/15–2020/3/20	Depression		PHQ-9	481(43.61)		4
Tu et al. (36)	China	100	21-46	2020/2/7–2020/2/25	Anxiety, depression	GAD-7	PHQ-9	40(40)	60(60)	6
Azoulay et al. (37)	France	845	NR	2020/10/30–2020/12/1	Anxiety, depression	HADS	HADS	507(60)	312(36.1)	4
Appel et al. (38)	Brazil	52	NR	2020/5–2020/7	Anxiety, depression	DASS-21	DASS-21	28(53.8)	20(38.4)	6
Zhang et al. (39)	China	276	NR	2020/3/5–2020/3/15	Anxiety, depression	HADS	HADS	77(27.9)	50(18.1)	5
Shen et al. (40)	China	643	20-56	2020/3/3–2020/3/10	Anxiety	GAD-7		215(33.4)		5
Li et al. (41)	China	150	NR	2020/2/1–2020/2/20	Anxiety, depression	HADS	HADS	59(39.3)	67(44.7)	8
Chalhub et al. (42)	Brazil	223	NR	2020/9–2020/10	Anxiety	BAI		38(17)		6
Bahadir-Yilmaz et al. (43)	Turkey	1,457	55	2020/4/25–2020/5/7	Anxiety	SAS		954(65.5)		6
Abu-Elenin (44)	Egypt	237	38.2 ± 6.2	2020/4–2020/5	Anxiety	GAD-7		187(78.9)		5
GebreEyesus et al. (45)	Ethiopia	322	NR	2020/11/10–2020/11/25	Anxiety, depression	GAD-7	PHQ-9	116(36)	83(25.8)	8
Barua et al. (46)	Bangladesh	370	30.5 ± 4.4	2020/4/1–2020/5/30	Anxiety, depression	GAD-2	PHQ-2	135(36.5)	142(38.4)	6
Kim and Yang (47)	Korea	180	31.5 ± 8.6	2020/6/10–2020/6/15	Anxiety, depression	DASS-21	DASS-21	84(46.7)	111(38.3)	5
Andrés-Olivera et al. (48)	Spain	77	25–62	2020/4/20–2020/4/26	Anxiety, depression	BAI	BDI-II	44(57.1)	40(53.3)	4

NR, not reported; SAS, Self-Rating Anxiety Scale; SDS, Self-Rating Depression Scale; DASS-21, Depression Anxiety Stress Scale 21; GAD-7, Generalized Anxiexy Disorde-7; PHQ-9, Patient Health Questionnaire-9; HADS, Hospital Anxiety and Depressive/Depression Scale; BAI, Beck Anxiety Inventory; BDI-II, Beck Depression Inventory-Second Edition.

**Table 2:** Results of quality assessment of included studies.

**Included studies**	**1**	**2**	**3**	**4**	**5**	**6**	**7**	**8**	**9**	**10**	**11**	**score**
Hu et al. (19)	Yes	Yes	Yes	Yes	No	Yes	No	Yes	No	Yes	No	7
Zhou et al. (20)	Yes	Yes	Yes	Yes	No	No	No	No	No	No	No	4
Li et al. (21)	Yes	Yes	Yes	Yes	No	No	Yes	No	No	Yes	No	6
Liu et al. (22)	Yes	Yes	Yes	Yes	No	No	Yes	No	No	Yes	No	6
Xu et al. (23)	Yes	Yes	Yes	Yes	No	No	No	No	No	Yes	No	5
Mei et al. (24)	Yes	Yes	No	Yes	No	No	No	No	No	Yes	No	4
Ma et al. (25)	Yes	Yes	No	Yes	No	No	No	No	No	Yes	No	4
Guo et al. (26)	Yes	Yes	Yes	Yes	No	Yes	Yes	Yes	No	Yes	No	8
Chu et al. (27)	Yes	Yes	Yes	Yes	No	No	Yes	Yes	No	Yes	No	7
Zhao et al. (28)	Yes	Yes	Yes	Yes	No	No	Yes	No	No	No	No	5
Tan et al. (29)	Yes	Yes	Yes	Yes	No	No	No	Yes	No	No	No	5
Yuan et al. (30)	Yes	Yes	No	Yes	No	Yes	Yes	Yes	No	Yes	No	7
Lei et al. (31)	Yes	Yes	Yes	Yes	No	No	Yes	No	No	No	No	5
Huang et al. (32)	Yes	Yes	Yes	Yes	No	No	No	No	No	Yes	No	5
Gong et al. (33)	Yes	Yes	Yes	Yes	No	No	No	No	No	No	No	4
Labrague and De Los Santos (34)	Yes	Yes	Yes	Yes	No	No	No	No	No	No	No	4
An et al. (35)	Yes	Yes	Yes	Yes	No	No	No	No	No	No	No	4
Tu et al. (36)	Yes	Yes	Yes	Yes	No	No	No	Yes	No	Yes	No	6
Azoulay et al. (37)	Yes	Yes	Yes	Yes	No	No	No	No	No	No	No	4
Appel et al. (38)	Yes	Yes	Yes	Yes	No	No	Yes	No	No	Yes	No	6
Zhang et al. (39)	Yes	Yes	Yes	Yes	No	No	No	No	No	Yes	No	5
Shen et al. (40)	Yes	Yes	Yes	Yes	No	No	No	Yes	No	No	No	5
Li et al. (41)	Yes	Yes	Yes	Yes	No	Yes	Yes	Yes	No	Yes	No	8
Chalhub et al. (42)	Yes	Yes	Yes	Yes	No	No	No	Yes	No	Yes	No	6
Bahadir-Yilmaz et al. (43)	Yes	Yes	Yes	Yes	No	No	Yes	Yes	No	No	No	6
Abu-Elenin (44)	Yes	Yes	Yes	Yes	No	No	No	No	No	Yes	No	5
GebreEyesus et al. (45)	Yes	Yes	Yes	Yes	No	Yes	Yes	Yes	No	Yes	No	8
Barua et al. (46)	Yes	Yes	Yes	Yes	No	No	Yes	No	No	Yes	No	6
Kim and Yang (47)	Yes	Yes	Yes	Yes	No	No	No	No	No	Yes	No	5
Andrés-Olivera et al. (48)	Yes	Yes	Yes	Yes	No	No	No	No	No	No	No	4

1, Define the source of information (survey, record review)? 2, List inclusion and exclusion criteria for exposed and unexposed subjects (cases and controls) or refer to previous publications? 3, Indicate time period used for identifying patients? 4, Indicate whether or not subjects were consecutive if not population-based? 5, Indicate if evaluators of subjective components of study were masked to other aspects of the status of the participants? 6, Describe any assessments undertaken for quality assurance purposes (e.g., test/retest of primary outcome measurements); 7, Explain any patient exclusions from analysis; 8, Describe how confounding was assessed and/or controlled; 9, If applicable, explain how missing data were handled in the analysis; 10, Summarize patient response rates and completeness of data collection; 11, Clarify what follow-up, if any, was expected and the percentage of patients for which incomplete data or follow-up was obtained.

### Meta-analysis results

#### Prevalence of anxiety

A total of 29 studies (19–34, 36–48) reported the prevalence of anxiety among frontline healthcare workers. The results showed that the overall prevalence of anxiety was 0.43, with a 95% CI (0.36, 0.50), *P* < 0.001. The results of subgroup analysis showed that among frontline healthcare workers, females [0.38, 95% CI (0.29, 0.48), *P* < 0.001], married individuals [0.39, 95% CI (0.26, 0.52), *P* < 0.001], those with children [0.51, 95% CI (0.47, 0.55), *P* < 0.001], those with a bachelor's degree or below [0.37, 95% CI (0.26, 0.48), *P* < 0.001], intermediate and above professional title [0.37, 95% CI (0.23, 0.52), *P* < 0.001], and nurses [0.45, 95% CI (0.32, 0.59), *P* < 0.001] had a higher prevalence of anxiety. Further details are provided in Table 3.

**Table 3:** Meta-analysis results of anxiety and depression among frontline healthcare workers during COVID-19.

**Outcomes**		**Number of included studies**	**Heterogeneity**		**Effect model**	**Prevalence (95% CI)**
			**test results**			
			***I*^2^(%)**	***P***		
**Anxiety**						
Overall prevalence		29 [19–34,36–48]	98.9	<0.001	Random	0.43 (0.36,0.50)
Gender	Male	11 [19,20,24,28,30,32,33,38,39,40,42]	90.0	<0.001	Random	0.26(0.16,0.35)
	Female	11 [19,20,24,28,30,32,33,38,39,40,42]	96.8	<0.001	Random	0.38 (0.29,0.48)
Marital status	Married	9 [19,20,24,28,30,32,38,40,42]	97.8	<0.001	Random	0.39 (0.26,0.52)
	single	9 [19,20,24,28,30,32,38,40,42]	92.1	<0.001	Random	0.34 (0.24,0.44)
Have children	Yes	5 [19,24,28,30,38]	24.3	0.260	Fixed	0.51 (0.47,0.55)
	No	5 [19,24,28,30,38]	43.8	0.130	Fixed	0.48 (0.43,0.53)
Educational level	Bachelor degree or below	10 [19,20,24,28,30,32,33,39,40,45]	93.2	<0.001	random	0.37 (0.26,0.48)
	Bachelor degree or above	10 [19,20,24,28,30,32,33,39,40,45]	97.0	<0.001	Random	0.34 (0.24,0.44)
Job title	Primary	7 [19,20,24,28,30,32,33]	96.9	<0.001	Random	0.37 (0.23,0.52)
	Intermediate and above	7 [19,20,24,28,30,32,33]	97.2	<0.001	Random	0.36(0.20,0.53)
Profession	doctor	6 [20,24,30,32,33,39]	92.4	<0.001	Random	0.25 (0.13,0.38)
	Nurse	11 [19,20,21,22,24,28,30,32,33,39,40]	98.4	<0.001	Random	0.45 (0.32,0.59)
**Depression**						
Overall prevalence		22 [20,21,23,31,33,35,39,41,45,48]	98.5	<0.001	Random	0.45 (0.37,0.52)
Gender	Male	8 [20,24,30,33,35,38,39,45]	92.6	<0.001	Random	0.37 (0.25,0.49)
	Female	8 [20,24,30,33,35,38,39,45]	97.5	<0.001	Random	0.40 (0.28,0.52)
Marital status	Married	5 [20,24,30,35,38]	98.4	<0.001	Random	0.47 (0.29,0.66)
	single	5 [20,24,30,35,38]	89.3	<0.001	Random	0.44 (0.33,0.56)
Have children	Yes	3 [24,30,38]	92.8	<0.001	Random	0.57 (0.34,0.81)
	No	3 [24,30,38]	76.3	0.015	Random	0.51 (0.33,0.69)
Educational level	Bachelor degree or below	7 [20,24,30,33,35,39,45]	79.2	<0.001	Random	0.33 (0.23,0.42)
	Bachelor degree or above	7 [20,24,30,33,35,39,45]	98.3	<0.001	Random	0.38 (0.24,0.51)
Job title	Primary	5 [20,24,30,33,35]	94.9	<0.001	Random	0.44 (0.33,0.55)
	Intermediate and above	5 [20,24,30,33,35]	98.5	<0.001	Random	0.48 (0.25,0.71)
Profession	Doctor	6 [20,24,30,33,39,45]	95.1	<0.001	Random	0.32 (0.15,0.48)
	Nurse	8 [20,21,24,30,33,35,39,45]	96.9	<0.001	Random	0.38 (0.27,0.49)

#### Prevalence of depression

A total of 22 studies (20, 21, 23–31, 35–39, 41, 45–48) reported the prevalence of depression among frontline healthcare workers. The results showed that the overall prevalence of depression was 0.45, with a 95% CI (0.37, 0.52), *P* < 0.001. The results of subgroup analysis showed that among frontline healthcare workers, females [0.40, 95% CI (0.28, 0.52), *P* < 0.001], married individuals [0.47, 95% CI (0.29, 0.66), *P* < 0.001], those with children [0.57, 95% CI (0.34, 0.81), *P* < 0.001], those with a bachelor's degree or above [0.38, 95% CI (0.24, 0.51), *P* < 0.001], intermediate and above professional title [0.48, 95% CI (0.25, 0.71), *P* < 0.001], and nurses [0.38, 95% CI (0.27, 0.49), *P* < 0.001] had a higher prevalence of depression. Further details are provided in Table 3.

### Sensitivity analysis

Sensitivity analysis was carried out by excluding the included studies individually. It was found that the prevalence of anxiety was 35–52%, and the prevalence of depression was 36–54%; the gap was not large compared to the overall prevalence rate. This suggests that the results of this study were stable.

### Publication bias analysis

The Egger test was used to evaluate publication bias, and the results showed that anxiety (*P* = 0.661) and depression (*P* = 0.266) all indicated that the possibility of publication bias was small.

## Discussion

Anxiety and depression are common but easily overlooked diseases (49). If patients with anxiety and depression are not properly treated, their quality of life may be affected. This study systematically evaluated the prevalence of anxiety and depression among frontline healthcare workers since the onset of COVID-19, which can be used to evaluate the impact of COVID-19 on their mental health. The results of the meta-analysis showed that the total prevalence of anxiety among frontline healthcare workers was 43% and the total prevalence of depression was 45%. The results of subgroup analysis showed that the prevalence of anxiety and depression in women, married individuals, those with children, and nurses was relatively high; the prevalence of anxiety was higher in those with a bachelor's degree or below, and the prevalence of depression was higher among those with intermediate professional titles and above.

This study reported that the overall prevalence of anxiety and depression among frontline healthcare workers during the COVID-19 pandemic was 43 and 45%, respectively, which was higher than the prevalence of anxiety (31.9%) and depression (33.7%) in the general population during the COVID-19 pandemic reported by Marvaldi et al. (50), which was also higher than the prevalence of anxiety (24.94%) and depression (24.83%) among healthcare workers during the COVID-19 pandemic reported by Sahebi et al. (13). In the early stages of the virus outbreak, lack of experience, and anti-pandemic materials, as well as long-term high-intensity work, may have caused higher rates of anxiety and depression among frontline healthcare workers than among the general population and healthcare workers. Simultaneously, frontline healthcare workers have a higher risk of infection, and the serious consequences of infection usually make people feel nervous and frightened. The physical and mental health of frontline healthcare workers becomes impaired, which in turn induces anxiety and depression. Lai et al. (51) also showed that the front-line work environment increases the risk of psychological problems for healthcare workers. Mental health quality levels should be properly assessed when selecting frontline healthcare workers. In addition, an existing study (52) found that direct exposure to COVID-19 also affected frontline healthcare workers' sleep quality. Good sleep quality protects against anxiety and depression. Therefore, providing adequate protective equipment and psychological support to frontline healthcare workers can improve their resilience. This will effectively reduce the risk of anxiety and depression, improve work status and efficiency, and provide favorable conditions for better anti-pandemic work. The prevalence of anxiety and depression has decreased with the normalization of infection prevention and control measures (53). Poor mental health can still have a negative impact on frontline healthcare workers. Therefore, it is necessary to regularly monitor the potential risks of anxiety and depression among frontline healthcare workers. This also provides effective interventions to improve their poor state, thereby reducing the related negative effects.

The results of the subgroup analysis showed that the prevalence of anxiety and depression was relatively high among women and nurses, which is consistent with the previous findings of Pappa (14). Previous studies (54) have shown that women are more likely to develop psychological disorders than men, which may be related to physiological factors such as hormone levels (55). Under normal circumstances, women's physical strength is not as great as that of men. When facing a long-term high-intensity workload, they may develop conflicted psychology, which may induce psychological problems, such as anxiety and depression. The prevalence of anxiety and depression among nurses is higher than that among doctors, which may be influenced by the fact that the majority of nurses are female. In addition, nurses spend more time in the ward providing direct care to patients than doctors and are more at risk of contracting the virus than doctors, so they are more prone to psychological problems. The prevalence of anxiety and depression among frontline healthcare workers who are married or have children is higher, which may be related to family burden (56). Owing to the special nature of their work, frontline healthcare workers may not be able to obtain information about their family members in a timely manner. Concerns about family cause them to devote part of their energy to worrying about the safety of their family members; therefore, psychological problems such as anxiety and depression may be more likely to occur.

This study's results are inconsistent regarding academic qualifications and professional titles. Changes in academic qualifications and professional titles had different effects on the prevalence of anxiety and depression, and further research is required. However, other studies (6) have proved that healthcare workers with lower professional titles and higher education have higher prevalence rates of anxiety and depression than those with higher professional titles and lower education, which provides a reference. Owing to the differences in the prevalence of anxiety and depression in different populations, we should formulate and provide corresponding preventive and intervention measures according to different types of needs.

This study has some limitations. First, the included studies were all cross-sectional, and some studies had small sample sizes. Second, the included studies were heterogeneous. Third, the number of included studies in some subgroups was small, which may have affected the results' accuracy to some extent.

In summary, since the onset of COVID-19, the prevalence of anxiety and depression among frontline healthcare workers has been relatively high, with obvious population differences. This group remains on the front line of the fight against the pandemic, and faces constant psychological pressure; therefore, attention should be paid to the anxiety and depression screening of frontline healthcare workers. Targeted measures should be taken to provide professional psychological intervention when necessary. Greater attention must now be paid to developing and evaluating the effectiveness of different interventions and initiatives to support the mental health of healthcare Workers during this pandemic (57). Simultaneously, domestic and international healthcare authorities and policymakers should take steps to reduce these mental illnesses among frontline healthcare workers. This could improve the efficiency of the hospital staff, accelerate pandemic control, and provide more effective treatments for COVID-19 patients.

Improving the medical security system, formulating targeted intervention measures, strengthening the training of healthcare workers, and providing professional psychological counseling can help improve mental health and avoid the emergence of serious psychological problems, such as anxiety and depression.

## Data availability statement

The datasets presented in this study can be found in online repositories. The names of the repository/repositories and accession numbers can be found in the article/supplementary materials.

## Author contributions

YuC conceived and designed the study and wrote the paper. YuC and JW collected data and conducted the follow-up work. YuC and YG performed the statistical analysis. ZF and LZ conducted the format and tables. YanC and YY directed the conduct of the study. All authors have read and approved the final manuscript.

## Funding

This work was supported by the Fifth Batch of Talents Selected under the Special Support Plan in Anhui Provence (Organization Department of Anhui Provincial Party Committee [2019] No. 14; T000516), Major Natural Science Research Projects in Universities of Anhui Province (No. KJ2020ZD69), and Anhui Province Social Science Innovation and Development Research Project (2021CX105).

## Conflict of interest

The authors declare that the research was conducted in the absence of any commercial or financial relationships that could be construed as a potential conflict of interest.

## Publisher's note

All claims expressed in this article are solely those of the authors and do not necessarily represent those of their affiliated organizations, or those of the publisher, the editors and the reviewers. Any product that may be evaluated in this article, or claim that may be made by its manufacturer, is not guaranteed or endorsed by the publisher.
